# Irisin response to acute moderate intensity exercise and high intensity interval training in youth of different obesity statuses: A randomized crossover trial

**DOI:** 10.14814/phy2.15198

**Published:** 2022-02-28

**Authors:** Benjamin H. Colpitts, Brittany V. Rioux, Ashley L. Eadie, Keith R. Brunt, Martin Sénéchal

**Affiliations:** ^1^ Cardiometabolic Exercise & Lifestyle Laboratory University of New Brunswick Fredericton New Brunswick Canada; ^2^ Faculty of Kinesiology University of New Brunswick Fredericton New Brunswick Canada; ^3^ Dalhousie Medicine New Brunswick Faculty of Medicine Department of Pharmacology Dalhousie University Saint John New Brunswick Canada; ^4^ IMPART Investigator Team Canada https://impart.team/

**Keywords:** adolescents, aerobic exercise, interval training, myokines, obesity

## Abstract

Limited data exist regarding the impact of an acute bout of exercise with varying intensities on irisin levels in the youth of different obesity statuses. The objectives were to (1) compare an acute bout of moderate continuous intensity (MCI) exercise and an acute bout of high‐intensity interval training (HIIT) on irisin response in youth with different obesity statuses and, (2) investigate whether changes in irisin levels are correlated with exploratory outcomes. A randomized crossover design study was conducted on 25 youth aged 12–18 years old. Participants were classified as either healthy weight (BMI percentile <85; *n* = 14) or overweight/obese (BMI percentile ≥85; *n* = 11). Participants performed an MCI exercise session at 50% of heart rate reserve for 35 min and a HIIT exercise session for 35 min, with intervals every 5 min increasing from 50% heart rate reserve to 85–90% for 2 min. Irisin was measured using an enzyme‐linked immunoabsorbent assay from plasma sampling obtained throughout the exercise (at times 0, 7, 14, 21, 28, and 35 min). A time effect was observed throughout the HIIT session [*F*(1,5) = 6.478, *p* < 0.001]. Bonferonni post‐hoc analysis revealed significant differences in irisin levels post‐exercise (35 min) compared to times 7, 14, 21, and 28 min. Irisin increased during HIIT (81.0% ± 71.3; *p* = 0.012) in youth with a healthy weight. No differences were observed for youth living as overweight or with obesity. Overall, HIIT elicits a higher peak irisin response compared to MCI exercise training in youth.

## Graphical abstract

High‐intensity interval training elicits a higher irisin response compared to moderate continuous‐intensity exercise in youth.

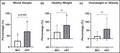

## 1 INTRODUCTION

Irisin is an adipo‐myokine primarily secreted by myocytes following skeletal muscle contractions (Boström et al., 2012). Muscle contractions lead to the activation of peroxisome ɣ and its coactivator‐1*α* (PGC‐1*α*) which; in turn, increases the expression of the fibronectin type III domain‐containing protein 5 (FNDC5) gene (Boström et al., 2012). The cleavage of this polypeptide protein at the N‐terminal is known as the hormone irisin. This hormone plays an integral role in energy metabolism by converting white fat to brown fat, which increases thermogenesis (Ahima & Park, 2015; Boström et al., 2012). Thus, irisin has been suggested as a potential mechanism for health benefits in individuals living with obesity and Type 2 diabetes.

Boström et al. (2012) observed a 2‐fold increase in plasma irisin following chronic aerobic exercise in adults, and others have corroborated these results (Hecksteden et al., 2013; Miyamoto‐Mikami et al., 2015). However, a meta‐analysis of chronic exercise training revealed a significant decrease in circulating irisin concentrations (Qiu et al., 2015). Interestingly though, in a recent meta‐analysis by our group, a 15% increase in circulating irisin concentrations was observed in adults following an acute bout of exercise (Fox et al., 2018). Although many studies have been published over recent years on irisin release during exercise, most of the available data is focused on adults, and little exists in youth. Research conducted by our team suggests that, in youth, acute aerobic exercise is associated with a significant increase in plasma irisin (29.23 ± 6.96 ng/ml vs. 39.30 ± 7.05 ng/ml; *p* = 0.028) (Blizzard LeBlanc et al., 2017). Others that have studied irisin in youth have found inconsistent results. For example, some authors suggest that plasma irisin increases post‐exercise in youth (Cai et al., 2019); however, others do not support such claims (Palacios‐González et al., 2015). Palacios‐Gonzáles et al. (2015) found no change in serum irisin concentrations following an 8‐month physical activity program in youth. Discrepancies between these studies could be explained by the complexity of exercise prescriptions, which might vary across studies in terms of supervision, mode, and intensity of exercise.

Studies performed in adults suggest that exercise intensity is an important factor to consider when investigating irisin's response to exercise. In these studies, a significant increase in irisin with high‐intensity interval training (HIIT) is obrserved as opposed to moderate continuous intensity (MCI) exercise (Eaton et al., 2018; Murawska‐Cialowicz et al., 2020; Rashti et al., 2019; Rezaeimanesh, 2020). To the best of our knowledge, the research of Archundia‐Herrera et al. (2017) is the sole study to investigate irisin release in youth following an acute bout of exercise of different intensities. This study observed a significant increase in skeletal muscle FNDC5 mRNA in adolescent females following HIIT as opposed to MCI exercise, while no difference was observed in the change of plasma irisin concentrations for HIIT (3.59 ng/ml ± 1.29 to 3.70 ng/ml ± 1.26) when compared to MCI exercise (3.66 ng/ml ± 0.80 to 3.56 ng/ml ±0.69; *p* = 0.62) (Archundia‐Herrera et al., 2017).

Therefore, the current data available on the impact of exercise on irisin response in youth are scarce. Since most data are from an adult population; did not provide data on the acute increase of irisin throughout the duration of the exercise session; and did not investigate irisin release in youth with different obesity statuses. The primary objective of this study was to investigate irisin release response during an acute bout of MCI exercise and HIIT in the youth of a healthy weight compared to those living as overweight or with obesity. The secondary objective was to investigate whether changes in irisin levels during exercise translate to changes in exploratory outcomes including metabolic profile, body composition, and cardiorespiratory fitness level.

## 2 MATERIALS AND METHODS

### 2.1 Study design and participant enrollment

**FIGURE 1:**
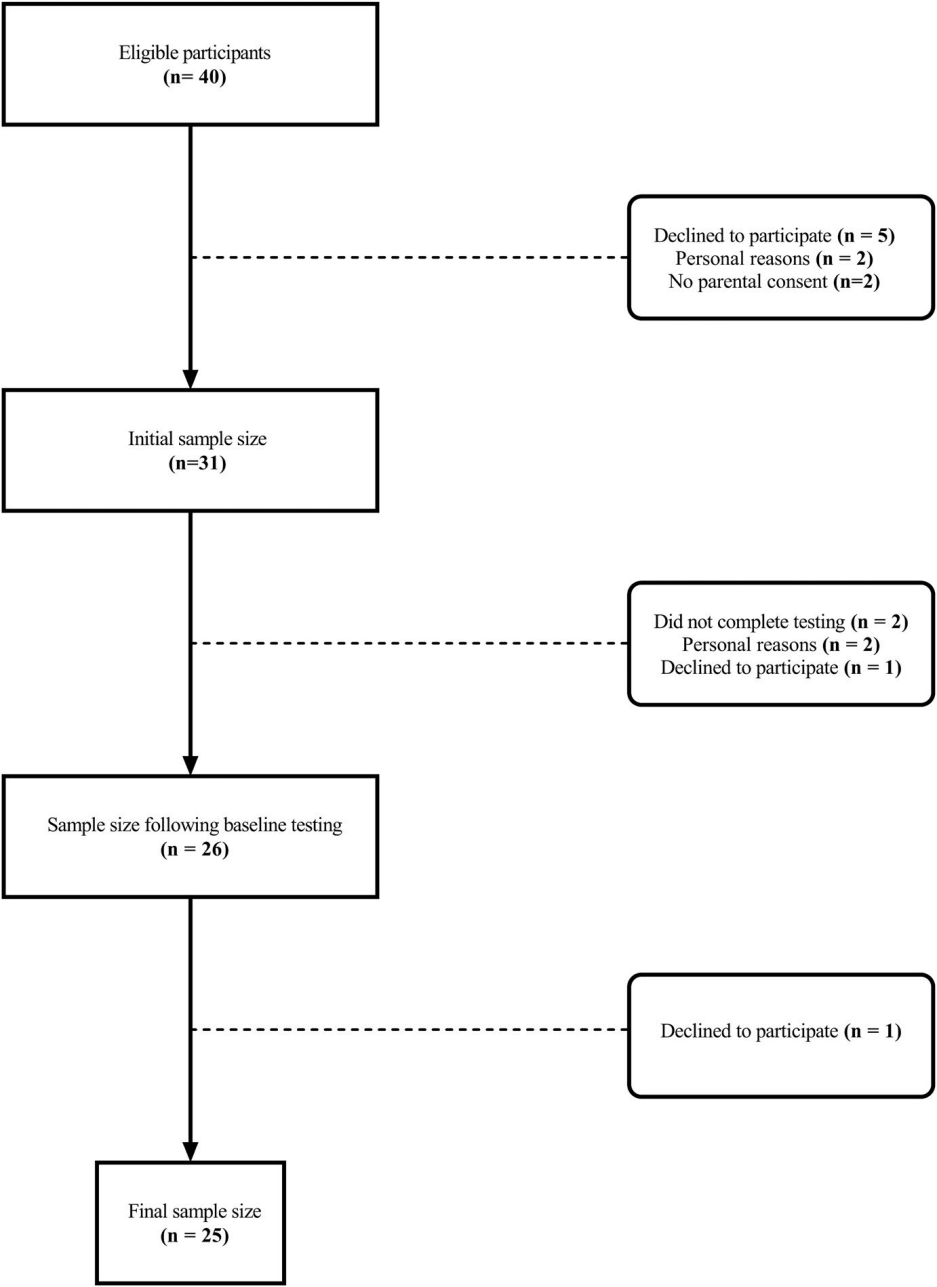
Participant flowchart

A randomized crossover design trial was performed to compare irisin responses during MCI exercise to HIIT in youth. Between September 2016 and August 2017, participants were recruited using social media platforms, newspapers, family physician clinics, and community events. Following pre‐screening on the phone, eligible participants were asked to come to the Cardiometabolic Exercise and Lifestyle Laboratory for additional screening and baseline testing. Overall, 40 youth were screened and deemed eligible for participation, and a total of 25 participants (13 males, 12 females) completed the study (Figure 1). All participants provided a consent form along with parental consent.

Participants were included in the study if they were: (1) Between 12 and 18 years old, and (2) had a body mass index (BMI) percentile either below the 85^th^ percentile for participants with a healthy weight, or for participants living as overweight or with obesity, a BMI equal to or above the 85^th^ percentile. Exclusion criteria included: (1) Taking any medication known to impact glucose metabolism, cause significant changes in weight, or be taking Metformin (which is known to impact irisin levels); (2) individuals living with a condition or injury that would impact their ability to perform the cycling sessions; and (3) individuals with glucose metabolism disorders, Type 2 diabetes mellitus or impaired glucose tolerance.

BMI was calculated using the following formula: Weight (kg)/height (m^2^). Height and weight measurements using the Canadian Society for Exercise Physiology (CSEP) protocol using a calibrated column scale (SECA^®^ model #213, Hamburg, Germany) (Canadian Society for Exercise Physiology, 2013). Briefly, height was measured following inhalation to the nearest 0.5 cm, while participants stood up straight with their feet together, arms to their side, and without shoes (Canadian Society for Exercise Physiology, 2013). For weight, the measurement was taken with minimal clothing and was measured to the nearest 0.1 kg (Canadian Society for Exercise Physiology, 2013). The according BMI was applied against the participant's age to determine the corresponding BMI zscore score, as per the *WHO Growth Charts for Canada* (World Health Organization, 2014a, 2014b).

### 2.2 Exposure variable—exercise intensity

**FIGURE 2:**
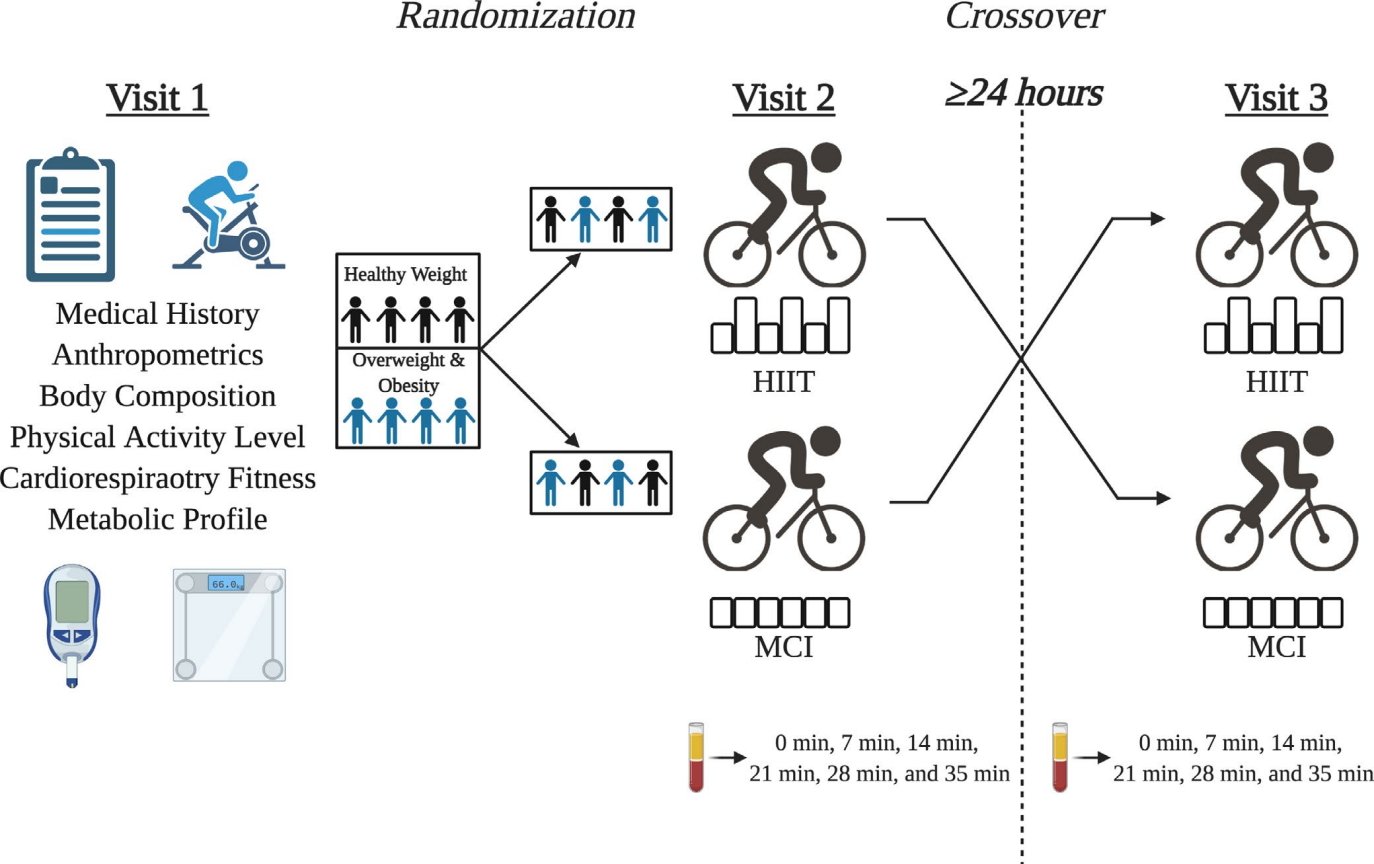
Experimental design & timeline. (Created with BioRender.com)

Following baseline testing, participants took part in two acute sessions of exercise training on a cycle ergometer: One MCI cycling session and one HIIT cycling session (Figure 2). The MCI cycling session involved 35 min of cycling at 50% heart rate reserve (HR_reserve_), while HIIT consisted of intervals at 50% HR_reserve_ for 5 min followed by 2 min of cycling at 85%–90% HR_reserve_. Five intervals were completed over a total of 35 min of cycling in the HIIT cycling session. HR_reserve_ was calculated using the following formula: HR_reserve_ = (HR_max_−HR_rest_) × %intensity−HR_rest_. Participants’ HR_rest_ was measured using the CSEP protocol (Canadian Society for Exercise Physiology, 2013). Briefly, participants were asked to sit and relax with their feet flat on the floor for 5 min (Canadian Society for Exercise Physiology, 2013). Then, heart rate was measured using a 15‐s count and was multiplied by four to determine beats per minute (Canadian Society for Exercise Physiology, 2013). Participants’ HR_max_ was determined using a VO_2max_ test. Heart rate was measured during the two acute sessions of exercise and VO_2max_ test using a Zephyr^TM^ BioHarness and OmniSense software (Medtronic, California, USA).

### 2.3 Outcome measure—plasma irisin concentration

Blood samples were collected intravenously by a registered nurse using a vacutainer coated with the anticoagulant ethylenediaminetetraacetic acid (EDTA), as recommended by the manufacturer. Blood draws were performed at the following timepoints: Pre‐exercise (0 min), and during the exercise at 7, 14, 21, 28, and immediately post‐exercise at 35 min. Blood samples were then centrifuged for 15 min at a spin rate of 16,000 g at 4°C to separate the plasma from the serum in the blood sample. Plasma was then collected and stored at −80 °C until further analysis. Irisin levels were measured at each time point using an Enzyme‐Linked Immunosorbent Assay (ELISA) from Phoenix Pharmaceuticals Inc. (EK‐067–29) per the manufacturer's protocol. The optical densities of the ELISA plate wells were measured using an EPOCH 2 Microplate Spectrophotometer (Biotek, Vermont, USA) with Gen5 software at a wavelength of 450 nm. Then, irisin concentrations were extrapolated from the optical density measurements using Prism version 8 (GraphPad Software, CA, USA).

### 2.4 Exploratory variables

#### 2.4.1 Body composition

Body composition was estimated using the BodPod version 1.69 (COSMED, California, USA). The BodPod is a reliable and validated tool that estimates body composition, with error scores in our lab being 3.5% for body fat percentage and 0.8% for fat‐free mass (Colpitts et al., 2021). Participants wore tight‐fitting clothing and a bathing cap while sitting still and breathing normally during the test. Based on participants’ body density value, fat‐free mass and fat mass were estimated using the Siri formula [% Body Fat = (495/Body Density) – 450] (Siri, 1993).

#### 2.4.2 Waist circumference

Waist circumference was measured using the CSEP protocol (Canadian Society for Exercise Physiology, 2013). The measurement was taken after a normal expiration at the upper lateral border of the iliac crest (Canadian Society for Exercise Physiology, 2013). Participants stood with their feet shoulder‐width apart and their arms crossed on their chest (Canadian Society for Exercise Physiology, 2013). An anthropometric tape measure was used for the measurement and waist circumference was recorded to the nearest 0.5 cm (Canadian Society for Exercise Physiology, 2013).

#### 2.4.3 Metabolic profile

Metabolic profile consisted of blood pressure, total cholesterol, HDL and LDL cholesterol, triglyceride levels, and glucose levels. Blood pressure was measured twice using the CSEP protocol (Canadian Society for Exercise Physiology, 2013). Participants were asked to sit for 5 min before taking each measurement (Canadian Society for Exercise Physiology, 2013). The average of the two measurements was used for resting blood pressure (Canadian Society for Exercise Physiology, 2013). Lipid and glucose profiles were obtained using the Cholestech LDX^®^ Analyzer (Alere Inc., Massachusetts, USA) following a 12‐h fast. An alcohol swab was used to clean the participant's left ring finger and was then dried off with a gauze pad (Cholestech LDX Analyzer, 2019). The finger was pricked with a lancet, then the tester gently compressed the finger to collect blood within the capillary tubes before transferring them into the cassette (Cholestech LDX Analyzer, 2019). The cassette was then inserted into the Cholestech LDX^®^ Analyzer for analysis. The Cholestech LDX^®^ Analyzer has been previously validated (Carey et al., 2006).

A single composite measure z‐score was created using two strategies. The first strategy included a multiple linear regression model with the individual variables of interest as outcome measures, with age and sex as independent variables. Since blood pressure is usually associated with height (Bourgeois et al., 2017), height was also included as a covariate in the models for systolic and diastolic blood pressure. Regression residuals were kept for all metabolic outcomes and used as individual cardiometabolic z‐scores. The second strategy consisted of the sum of all of the measured cardiometabolic risk factors to create a single cardiometabolic z‐score. To account for the inverse relationship between HDL‐cholesterol and cardiovascular risk, HDL cholesterol residuals were multiplied by a negative constant (−1) before being added to the composite score, as done previously (Rioux et al., 2017).

#### 2.4.4 Cardiorespiratory fitness

Cardiorespiratory fitness was measured by performing a graded cycle ergometer test using a TrueMax 2400 Metabolic Measurement Cart (ParvoMedics, Utah, USA). Participants were instructed to pedal at a cadence of 60–65 revolutions per minute. Participants began cycling at 25 Watts and the wattage was increased by 25 watts every 2 min until a respiratory exchange ratio (RER) of 1.00 was obtained by the participant. Then, the resistance was increased by 25 Watts per minute until participant exhaustion, as before (Sénéchal et al., 2013, 2015). The average of the highest six consecutive VO_2_ values (using 5‐second averages) was used to estimate the participants’ VO_2peak_ score. Maximum heart rate and RER were also recorded.

### 2.5 Statistical analysis

Baseline characteristics are presented as mean (SD) for continuous variables and N (%) for categorical variables unless otherwise stated. Shapiro‐Wilk tests were used to test for normality, and visual exploration of the data was performed to further investigate the normality of the distribution. *Repeated measures analyses of variance (ANOVAs)* were performed to investigate (1) the interaction between time and exercise mode on irisin release, and (2) the interaction between time and obesity status on irisin release. Bonferroni posthoc analyses were used to identify any timepoint differences in irisin concentrations. *Independent sample t*‐*test*s were performed to determine group differences in baseline measurements. *Paired sample t*‐*tests* were performed to determine whether there was a significant difference between baseline irisin levels (time 0 min) and peak irisin levels, as well as differences between percent change in irisin levels per condition and group. *One sample t*‐*tests* were used to determine whether there was a significant difference between the percent change of irisin throughout the exercise (0 min to peak irisin levels) in comparison to 0% change per condition and group. *Bivariate Pearson's correlations* were performed to investigate if changes in irisin concentration were associated with exploratory outcomes. Data management and statistical analyses were performed using SPSS version 16. A *p* ≤ 0.05 was considered significant.

## 3 RESULTS

No difference was observed in the proportion of males between the participants with healthy body weight (50%) and those living as overweight or with obesity (54.6%; *p* = 0.830). Participants living as overweight or with obesity had a significantly higher fat mass [25.46 kg (SD 15.68) vs. 11.55 kg (SD 4.91); *p* = 0.005] compared to participants with a healthy weight, while no significant difference was observed for fat‐free mass (*p* = 0.526) between groups (Table 1). Participants with a healthy weight had significantly higher cardiorespiratory fitness [44.18 ml/kg/min (SD 8.75)] compared to those living as overweight or with obesity [34.80 ml/kg/min (SD 7.24); *p* = 0.009]. No difference in baseline irisin levels was observed between obesity groups for either condition (all *p* > 0.05).

**TABLE 1:** Baseline characteristics of the sample stratified by obesity status

	Healthy weight *n* = 14	Overweight or obesity *n* = 11	*p*
Age (years)	17.14 ± 1.66	16.27 ± 2.05	0.253
Sex (% males)	7 (50.0)	6 (54.5)	0.830
Anthropometrics			
Weight (kg)	62.35 ± 11.93	79.15 ± 15.45	**0.005**
BMI (kg/m^2^)	20.30 ± 2.19	25.29 ± 2.83	**< 0.001**
BMI Percentile	48.57 ± 19.98	90.64 ± 6.50	**< 0.001**
BMI z‐score	−0.21 ± 0.65	1.31 ± 0.57	**< 0.001**
Waist circumference (cm)	79.02 ± 11.85	88.16 ± 13.96	0.090
Fat mass (kg)	11.55 ± 4.91	25.46 ± 15.68	**0.005**
Fat mass (%)	19.09 ± 8.34	28.15 ± 10.95	**0.027**
Fat‐free mass (kg)	55.39 ± 14.42	59.09 ± 14.08	0.526
Metabolic profile			
Systolic BP (mmHg)	111.70 ± 15.05	117.10 ± 15.14	0.386
Diastolic BP (mmHg)	71.89 ± 7.65	75.14 ± 6.47	0.273
Total cholesterol (mmol/L)	4.38 ± 0.84	4.31 ± 0.84	0.851
HDL cholesterol (mmol/L)	1.57 ± 0.32	1.48 ± 0.51	0.599
LDL cholesterol (mmol/L)	2.43 ± 0.88	2.52 ± 0.87	0.808
Triglycerides (mmol/L)	1.01 ± 0.40	1.07 ± 0.56	0.778
Glucose (mmol/L)	5.03 ± 0.40	5.31 ± 0.45	0.123
Metabolic z‐score	−0.67 ± 1.69	0.86 ± 2.48	0.080
Fitness and activity levels			
CRF (ml/kg/min)	44.18 ± 8.75	34.80 ± 7.24	**0.009**
CRF (ml/FFM/min)	51.46 ± 12.47	47.40 ± 9.78	0.386
Baseline irisin concentrations			
MCI (ng/ml)	20.65 ± 8.05	20.34 ± 5.68	0.917
HIIT (ng/ml)	19.32 ± 13.16	16.23 ± 9.47	0.533

Note

Continuous variables are presented as means ± standard deviation. Categorical variables are presented as *n* (%). Bolded values represent a significant difference between groups; alpha level at 0.05.

Abbreviations: BMI, body mass index; BP, blood pressure; CRF, cardiorespiratory fitness; HDL, high density lipoprotein; HIIT, high intensity interval training; LDL, low density lipoprotein; MCI, moderate continuous intensity; MVPA, moderate‐to‐vigorous physical activity.

**FIGURE 3:**
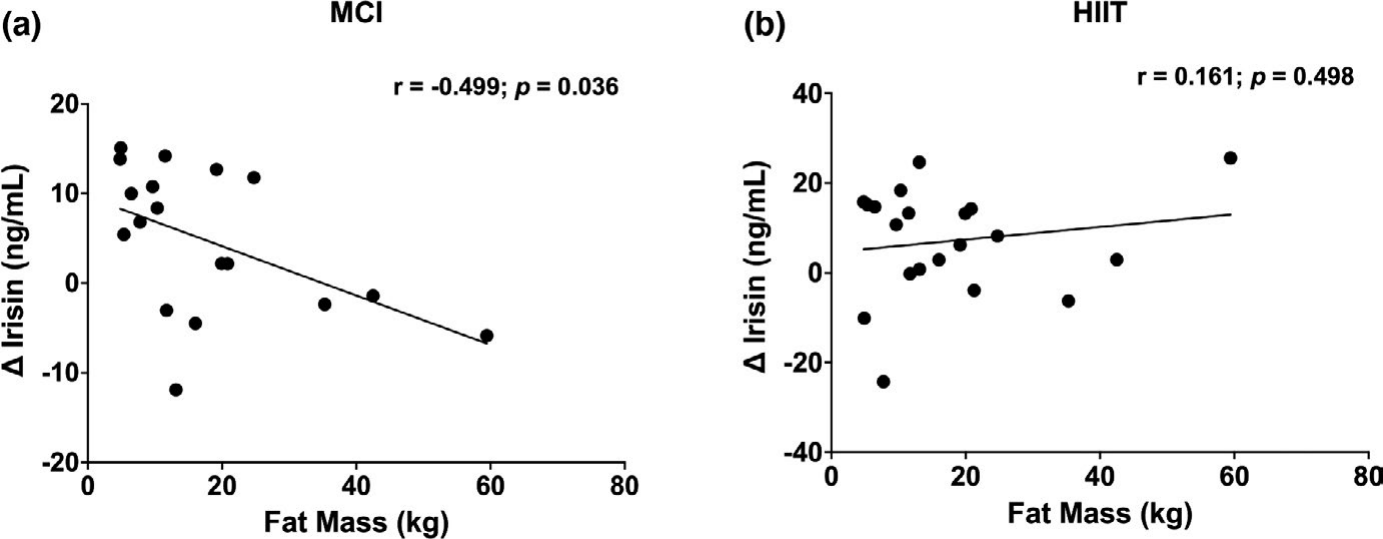
(a, b) Association between change in irisin concentration (baseline to peak) during both exercise sessions and fat mass for the whole sample. Alpha level at 0.05

**FIGURE 4:**
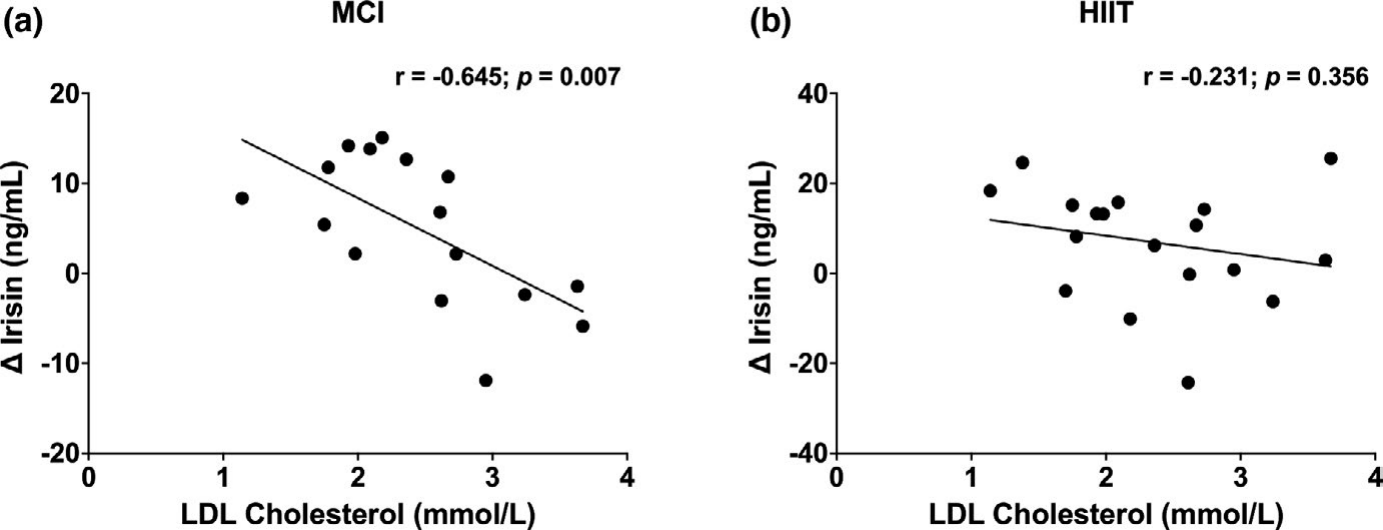
(a, b) Association between change in irisin concentration (baseline to peak) during both exercise sessions and LDL cholesterol for the whole sample. Alpha level at 0.05

Change in irisin (between baseline and peak irisin) was negatively correlated with total fat mass (−0.499; *p* = 0.036; Figure 3a) and LDL cholesterol (−0.645; *p* = 0.007; Figure 4a) in MCI exercise while no such results were observed for HIIT (Figures 3b and 4b). No correlations were observed between the change in irisin and other exploratory variables (all *p* > 0.05; Table 2).

**TABLE 2:** Pearson correlations between the change in plasma irisin and exploratory measures for the whole sample

	MCI T0‐Peak	HIIT T0‐Peak
Age (years)	−0.035	−0.288
Weight (kg)	−0.290	−0.233
BMI (kg/m^2^)	−0.126	−0.095
Waist circumference (cm)	−0.397	−0.167
Fat mass (%)	−0.401	0.055
Fat‐free mass (kg)	0.255	−0.181
Systolic BP (mmHg)	−0.106	−0.330
Diastolic BP (mmHg)	−0.324	−0.264
Total cholesterol (mmol/L)	−0.463	−0.255
HDL cholesterol (mmol/L)	0.235	0.022
Triglycerides (mmol/L)	−0.234	−0.205
Glucose (mmol/L)	0.190	−0.021
Metabolic z‐scores	−0.071	0.144
CRF (ml/kg/min)	0.091	−0.183

Note

Data presented as Pearson correlations. Bolded values represent significant correlation; alpha level at 0.05.

Abbreviations: BMI, body mass index; BP, blood pressure; CRF, cardiorespiratory fitness; HDL, high density lipoprotein; HIIT, high intensity interval training T0, timepoint 0 min; MCI, moderate continuous intensity; MVPA, moderate‐to‐vigorous physical activity.

**FIGURE 5:**
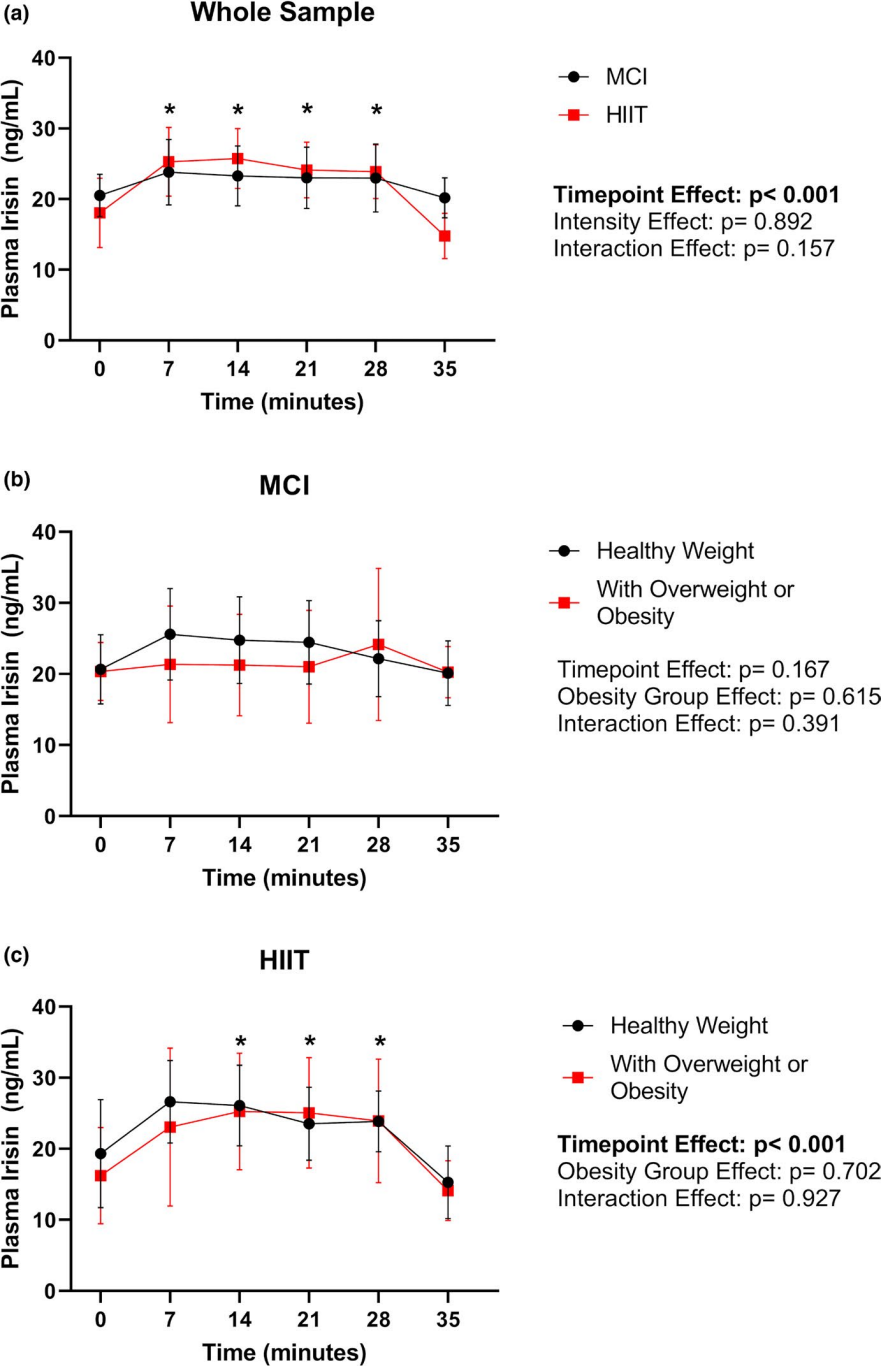
(a, b, c) Plasma irisin levels throughout the exercise bouts for the whole sample (stratified by exercise intensity) and for each exercise intensity condition (stratified by obesity status). Data presented as means and 95% confidence intervals. MCI, moderate continuous intensity; HIIT, high intensity interval training. Alpha level at 0.05. * represents a significant difference from time point 35 min

Figure 5a–c present the results from the repeated measures ANOVAs that explored differences in irisin concentration across time points for the whole sample, stratified by the exercise intensity, and for both exercise sessions stratified by obesity status. A significant time effect was observed when looking at the whole sample [*F*(1,5) = 7.812, *p* < 0.001; Figure 5a]. Bonferonni posthoc analyses revealed significant differences in irisin concentration between 35 min and 7, 14, 21, and 28 min (all *p* < 0.05). Irisin concentration was not significantly different between any of the time points during the MCI exercise session (Figure 5b). However, a significant time effect was observed for the HIIT session [*F*(1,5) = 6.478, *p* < 0.001; Figure 5c]. Bonferonni posthoc analyses revealed significant differences in irisin concentration between 35 min and 14, 21, and 28 min of HIIT (all *p* < 0.05).

**FIGURE 6:**
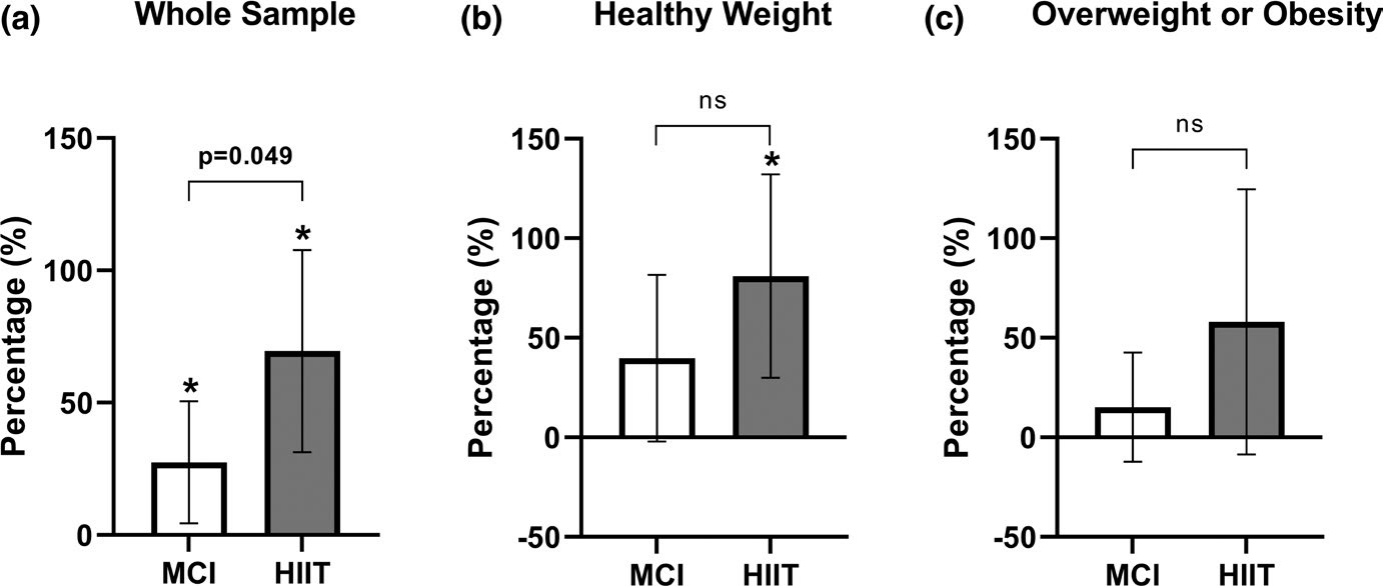
(a, b, c) Percent change in plasma irisin levels from baseline to peak for the whole sample and stratified by obesity status. Data presented as mean and 95% confidence interval. MCI, moderate continuous intensity; HIIT, high intensity interval training. Alpha level at 0.05. * represents a significant change within the condition

Figure 6a–c present the percent change in irisin concentration from baseline to peak irisin levels for the whole sample and stratified by obesity status. For the whole sample, percent change in irisin during the MCI exercise and HIIT sessions were significantly different (*p* = 0.049; Figure 6a), with irisin concentration increasing in both the MCI exercise [27.5% (SD 46.3), *p* = 0.022] and HIIT sessions [69.5% (SD 81.6), *p* = 0.001]. A significant increase in peak irisin was observed for the HIIT session [57.4% (SD 72.0); *p* = 0.012]; however, this was not significantly different when compared to the MCI exercise session [38.4% (SD 54.1), *p* = 0.272; Figure 6b] in youth with a healthy weight. For youth living as overweight or with obesity, no significant difference in the percent change in irisin was observed between MCI exercise and HIIT (*p* = 0.079; Figure 6c).

## 4 DISCUSSION

The main objective of this study was to investigate the changes in irisin during an acute bout of MCI exercise and HIIT using a randomized crossover design comparing youth living as overweight or with obesity to youth with a healthy weight. Our main findings suggest that: (1) Significant differences in irisin concentrations were observed over time during an acute bout of HIIT, (2) significant differences in percent change in irisin concentrations were observed between exercise conditions, (3) youth with a healthy weight elicited an increase in irisin concentration during HIIT session, whereas those living as overweight or with obesity did not have a significant change in irisin during exercise, and (4) fat mass and LDL cholesterol were negatively correlated with irisin release. These findings provide further insight on the impact of exercise intensity on irisin release in the youth of different obesity statuses and provide relevant information for the management of obesity and obesity‐related cardiometabolic risk factors in youth.

### 4.1 Eliciting irisin bioavailability in youth living as overweight or with obesity

A significant time effect was observed for change in irisin over the course of 35 min of aerobic exercise in the whole sample of youth. In addition, when stratifying these analyses per condition, we found a time effect only for HIIT, while no such results were observed for MCI. These results align with previous data (Huh et al., 2015; Kim et al., 2016) showing that aerobic exercise increases the release of irisin. More importantly, this result is in accordance with a meta‐analysis published by our group, suggesting that acute exercise leads to increases in irisin release (Fox et al., 2018). We build on pre‐existing data of irisin metabolism and exercise by documenting the irisin response in youth. A time effect was observed, but only in the HIIT group, which is in line with previous findings performed in adults (Archundia‐Herrera et al., 2017; Daskalopoulou et al., 2014; Tsuchiya et al., 2014) that show irisin increases in a dose‐response manner with an intensity of aerobic exercise. However, Winn et al. (2017) investigated irisin levels pre‐exercise and 30, 50, 80, and 190 min into both MCI exercise and HIIT in female adults living with obesity and observed peak irisin levels at 50 min for both exercise intensities. Having said that, it is possible that a time effect may have been observed in the MCI exercise session as well if we analyzed irisin concentrations post‐exercise.

### 4.2 Potential setpoint alteration of irisin amongst youth living as overweight or with obesity

Interestingly, when looking at the whole cohort of youth, irisin levels significantly increased from baseline to peak in both exercise conditions—with HIIT displaying the greatest change. BMI stratified analyses revealed that youth with a healthy weight displayed a significant increase in irisin concentration when performing HIIT; however, no such difference was observed for the MCI exercise session. This is consistent with previous findings from Huh et al. (2014), who observed a significant increase in irisin concentrations following HIIT swimming in adolescents; however, no increase was observed during a bout of MCI swimming (Huh et al., 2014). These findings suggests that exercise intensity plays a key role in irisin release, specifically in youth. In the current trial, no significant change was observed in youth living as overweight or with obesity. This lack of change could be explained by methodological differences in comparison to previous literature. For example, Blizzard‐LeBlanc et al. (2017) observed a 60% increase in irisin release following 45 min of MCI aerobic exercise at 60% of heart rate reserve (29.23 ng/ml ±6.96–39.30 ng/ml ±7.05; *p* = 0.028). Although, the BMI classification for obesity between studies was different; the present study included both youth living as overweight or with obesity (≥85^th^ percentile), whereas the former study investigated those solely living with obesity (≥95^th^ percentile) (Blizzard LeBlanc et al., 2017). Additionally, their exercise intensity was slightly higher and exercise duration was 10 min longer (Blizzard LeBlanc et al., 2017), which may impact irisin release. Furthermore, Archundia‐Herrera et al. (2017) observed a significant increase in muscle irisin levels following HIIT in adolescent females while they did not report any changes following MCI exercise. However, they did not observe differences in plasma irisin release between conditions (Archundia‐Herrera et al., 2017). Together, our findings add to the existing literature on the impact of exercise intensity on irisin release in youth, suggesting that HIIT may elicit higher levels of irisin release, especially in youth with a healthy weight.

### 4.3 Risk factors associated with participant's irisin bio‐availability

In the current study, change in irisin was negatively correlated with fat mass and LDL cholesterol. The relationship between irisin and fat mass has been extensively researched in adults (Elizondo‐Montemayor et al., 2019; Pardo et al., 2014; Zhang et al., 2020). However, these studies are limited to the analysis of irisin release with chronic exercise interventions and few on acute exercise interventions, reporting only resting irisin measures (without the change during exercise), and inconsistent results. For example, a cross‐sectional analysis revealed that youth living with obesity had higher irisin levels at rest [141.2 ng/ml, (IQR 90.1–195.9)] compared to a healthy weight control group [107.6 ng/ml, (IQR 89.5–138.3); *p* = 0.024] (Çatlı et al., 2016). These findings, in combination with the current trial, suggests that fat mass may be a key mediator in terms of irisin release in youth. The relationship between irisin and LDL cholesterol has been inconsistent in the literature, with findings differing according to the population studied and age group. In both De Meneck et al.’s (2018) and Shim et al. (2018) analyses, no relationship was observed between LDL cholesterol and irisin in children living as overweight or with obesity, respectively. This was similar to our analysis, as when stratifying per BMI category, no association was found between LDL cholesterol and irisin in those living as overweight or with obesity (results not shown). However, an inverse relationship between the two variables emerged when analyzing the entire cohort of youth in our study, which has also been documented in adults (Oelmann et al., 2016). Altogether, these findings suggest that LDL cholesterol may play a role in irisin release in youth, regardless of BMI classification.

### 4.4 Strengths and limitations

Although our study provides novel insight into the impact of exercise intensity on irisin release in the youth of different obesity status, some limitations should be noted. First, the exclusion of a non‐exercising control condition limits the inferences that can be made with our findings. Likewise, the inclusion of important clinical health measures (such as insulin resistance) would have provided increasingly relevant information on their association with irisin release. The exclusion of a post‐exercise irisin measurement eliminated our ability to explore whether irisin release peaked following the completion of exercise. Nevertheless, the current study is strengthened by being the first to compare irisin release during exercise in both youth living as overweight or with obesity and in those with a healthy weight. Additionally, the nature of both the primary exposure and outcome measure was strictly controlled for by the use of heart rate reserve for the HIIT and MCI conditions.

## 5 CONCLUSION

Our findings suggest differences exist in irisin release between the two exercise conditions in youth. More specifically, HIIT elicits a higher peak irisin concentration compared to MCI exercise; specifically in healthy weight youth as opposed to youth living as overweight or with obesity. Future research should focus on relating irisin release to other health outcomes and investigating the time in which irisin release peaks during various exercise modalities.

## CONFLICT OF INTEREST

The authors have no conflict of interest to declare.

## ETHICAL APPROVAL

The study was approved by the University of New Brunswick Research Ethics Board (REB File #2016‐099).

## AUTHOR CONTRIBUTIONS

Martin Sénéchal conceived and designed the research study. Brittany V. Rioux, Ashley L. Eadie, Keith R. Brunt, and Martin Sénéchal performed experiments and contributed to data collection. Benjamin H. Colpitts and Martin Sénéchal contributed to data analysis and preparation of figures. Benjamin H. Colpitts, Brittany V. Rioux, and Martin Sénéchal contributed to the interpretation of the results and drafting the manuscript. Benjamin H. Colpitts, Brittany V. Rioux, Ashley L. Eadie, Keith R. Brunt, and Martin Sénéchal contributed to editing and revising the manuscript as well as proving final approval of the manuscript. The manuscript and figures were not published elsewhere.

## References

[phy215198-bib-0001] Ahima, R. S. , & Park, H.‐K. (2015). Connecting myokines and metabolism. Endocrinology and Metabolism, 30(3), 235–245. 10.3803/EnM.2015.30.3.235 26248861 PMC4595346

[phy215198-bib-0002] Archundia‐Herrera, C. , Macias‐Cervantes, M. , Ruiz‐Muñoz, B. , Vargas‐Ortiz, K. , Kornhauser, C. , & Perez‐Vazquez, V. (2017). Muscle irisin response to aerobic vs HIIT in overweight female adolescents. Diabetology & Metabolic Syndrome, 9, 101. 10.1186/s13098-017-0302-5 29299068 PMC5746008

[phy215198-bib-0003] Blizzard LeBlanc, D. R. , Rioux, B. V. , Pelech, C. , Moffatt, T. L. , Kimber, D. E. , Duhamel, T. A. , Dolinsky, V. W. , McGavock, J. M. , & Sénéchal, M. (2017). Exercise‐induced irisin release as a determinant of the metabolic response to exercise training in obese youth: The EXIT trial. Physiological Reports, 5(23), e13539. 10.14814/phy2.13539 PMC5727287 29208692

[phy215198-bib-0004] Boström, P. , Wu, J. , Jedrychowski, M. P. , Korde, A. , Ye, L. , Lo, J. C. , Rasbach, K. A. , Boström, E. A. , Choi, J. H. , Long, J. Z. , Kajimura, S. , Zingaretti, M. C. , Vind, B. F. , Cinti, H. T. S. , Højlund, K. , Gygi, S. P. , & Spiegelman, B. M. (2012). A PGC1‐α‐dependent myokine that drives brown‐fat‐like development of white fat and thermogenesis. Nature, 481(7382), 463–468. 10.1038/nature10777 22237023 PMC3522098

[phy215198-bib-0005] Bourgeois, B. , Watts, K. , Thomas, D. M. , Carmichael, O. , Hu, F. B. , Heo, M. , Hall, J. E. , & Heymsfield, S. B. (2017). Associations between height and blood pressure in the United States population. Medicine (Baltimore), 96(50), e9233. 10.1097/MD.0000000000009233 29390353 PMC5815765

[phy215198-bib-0006] Cai, L. , Tan, M. , Tan, W. , Zeng, X. , Wan, N. , Wong, S. H. , O'Reilly, J. , Sun, F. , Yang, J. , & Chen, Y. (2019). Associations of circulating irisin concentrations with cardiometabolic risk factors among children vary by physical activity or sedentary time levels. Frontiers in Endocrinology, 10, 549. 10.3389/fendo.2019.00549 31474938 PMC6703142

[phy215198-bib-0007] Carey, M. , Markham, C. , Gaffney, P. , Boran, C. , & Maher, V. (2006). Validation of a point of care lipid analyser using a hospital based reference laboratory. Irish Journal of Medical Science, 175(4), 30–35. 10.1007/BF03167964 17312826

[phy215198-bib-0008] Çatlı, G. , Küme, T. , Tuhan, H. Ü. , Anık, A. , Çalan, Ö. G. , Böber, E. , & Abacı, A. (2016). Relation of serum irisin level with metabolic and antropometric parameters in obese children. Journal of Diabetes and Its Complications, 30(8), 1560–1565. 10.1016/j.jdiacomp.2016.07.019 27539885

[phy215198-bib-0009] Cholestech LDX Analyzer . (2019). Lipid Profile, Cholesterol, and Glucose Testing. ‐ Alere is now Abbott [Internet]. [cited 2019 December 3]. Available from: https://www.alere.com/en/home/product‐details/cholestech‐ldx‐system.html

[phy215198-bib-0010] Colpitts, B. H. , Seaman, K. , Bouchard, D. R. , & Sénéchal, M. (2021). Difference in total workload during sprint interval training for adults living with or without obesity. European Journal of Applied Physiology, 121(10), 2893–2902. 10.1007/s00421-021-04760-y 34191095

[phy215198-bib-0011] Daskalopoulou, S. S. , Cooke, A. B. , Gomez, Y.‐H. , Mutter, A. F. , Filippaios, A. , Mesfum, E. T. , & Mantzoros, C. S. (2014). Plasma irisin levels progressively increase in response to increasing exercise workloads in young, healthy, active subjects. European Journal of Endocrinology, 171(3), 343–352. 10.1530/EJE-14-0204 24920292

[phy215198-bib-0012] De Meneck, F. , Victorino de Souza, L. , Oliveira, V. , & do Franco, M. C. (2018). High irisin levels in overweight/obese children and its positive correlation with metabolic profile, blood pressure, and endothelial progenitor cells. Nutrition, Metabolism and Cardiovascular Diseases, 28(7), 756–764. 10.1016/j.numecd.2018.04.009 29858156

[phy215198-bib-0013] Eaton, M. , Granata, C. , Barry, J. , Safdar, A. , Bishop, D. , & Little, J. P. (2018). Impact of a single bout of high‐intensity interval exercise and short‐term interval training on interleukin‐6, FNDC5, and METRNL mRNA expression in human skeletal muscle. Journal of Sport and Health Science, 7(2), 191–196. 10.1016/j.jshs.2017.01.003 30356443 PMC6180539

[phy215198-bib-0014] Elizondo‐Montemayor, L. , Gonzalez‐Gil, A. M. , Tamez‐Rivera, O. , Toledo‐Salinas, C. , Peschard‐Franco, M. , Rodríguez‐Gutiérrez, N. A. , Silva‐Platas, C. , & Garcia‐Rivas, G. (2019). Association between Irisin, hs‐CRP, and metabolic status in children and adolescents with type 2 diabetes mellitus. Mediators of Inflammation, 2019, e6737318. 10.1155/2019/6737318 PMC6446111 31015797

[phy215198-bib-0015] Fox, J. , Rioux, B. V. , Goulet, E. D. B. , Johanssen, N. M. , Swift, D. L. , Bouchard, D. R. , Loewen, H. , & Sénéchal, M. (2018). Effect of an acute exercise bout on immediate post‐exercise irisin concentration in adults: A meta‐analysis. Scandinavian Journal of Medicine and Science in Sports, 28(1), 16–28. 10.1111/sms.12904 28453881

[phy215198-bib-0016] Canadian Society for Exercise Physiology . (2013). CSEP‐PATH: Physical activity training for health.

[phy215198-bib-0017] Hecksteden, A. , Wegmann, M. , Steffen, A. , Kraushaar, J. , Morsch, A. , Ruppenthal, S. , Kaestner, L. , & Meyer, T. (2013). Irisin and exercise training in humans ‐ results from a randomized controlled training trial. BMC Medicine, 11, 235. 10.1186/1741-7015-11-235 24191966 PMC4228275

[phy215198-bib-0018] Huh, J. Y. , Mougios, V. , Kabasakalis, A. , Fatouros, I. , Siopi, A. , Douroudos, I. I. , Filippaios, A. , Panagiotou, G. , Park, K. H. , & Matzoros, C. S. (2014). Exercise‐induced irisin secretion is independent of age or fitness level and increased irisin may directly modulate muscle metabolism through AMPK activation. Journal of Clinical Endocrinology and Metabolism, 99(11), E2154–E2161. 10.1210/jc.2014-1437 25119310

[phy215198-bib-0019] Huh, J. Y. , Siopi, A. , Mougios, V. , Park, K. H. , & Mantzoros, C. S. (2015). Irisin in response to exercise in humans with and without metabolic syndrome. Journal of Clinical Endocrinology and Metabolism, 100(3), E453–457. 10.1210/jc.2014-2416 25514098

[phy215198-bib-0020] Kim, H.‐J. , Lee, H.‐J. , So, B. , Son, J. S. , Yoon, D. , & Song, W. (2016). Effect of aerobic training and resistance training on circulating irisin level and their association with change of body composition in overweight/obese adults: A pilot study. Physiological Research, 65(2), 271–279. 26447516 10.33549/physiolres.932997

[phy215198-bib-0021] Miyamoto‐Mikami, E. , Sato, K. , Kurihara, T. , Hasegawa, N. , Fujie, S. , Fujita, S. , Sanada, K. , Hamaoka, T. , Tabata, I. , & Iemitsu, M. (2015). Endurance training‐induced increase in circulating irisin levels is associated with reduction of abdominal visceral fat in middle‐aged and older adults. PLoS One, 10(3), e0120354. 10.1371/journal.pone.0120354 25793753 PMC4368602

[phy215198-bib-0022] Murawska‐Cialowicz, E. , Wolanski, P. , Zuwala‐Jagiello, J. , Feito, Y. , Petr, M. , Kokstejn, J. , Stastny, P. , & Goliński, D. (2020). Effect of HIIT with tabata protocol on serum irisin, physical performance, and body composition in men. International Journal of Environmental Research and Public Health, 17(10), 3589. 10.3390/ijerph17103589 PMC7277607 32443802

[phy215198-bib-0023] Oelmann, S. , Nauck, M. , Völzke, H. , Bahls, M. , & Friedrich, N. (2016). Circulating irisin concentrations are associated with a favourable lipid profile in the general population. PLoS One, 11(4), e0154319. 10.1371/journal.pone.0154319 27128661 PMC4851367

[phy215198-bib-0024] Palacios‐González, B. , Vadillo‐Ortega, F. , Polo‐Oteyza, E. , Sánchez, T. , Ancira‐Moreno, M. , Romero‐Hidalgo, S. , Meráz, N. , & Antuna‐Puente, B. (2015). Irisin levels before and after physical activity among school‐age children with different BMI: A direct relation with leptin. Obesity, 23(4), 729–732. 10.1002/oby.21029 25820255

[phy215198-bib-0025] Pardo, M. , Crujeiras, A. B. , Amil, M. , Aguera, Z. , Jiménez‐Murcia, S. , Baños, R. , Botella, C. , de la Torre, R. , Estivill, X. , Fagundo, A. B. , Fernández‐Real, J. M. , Fernández‐García, J. C. , Fruhbeck, G. , Gómez‐Ambrosi, J. , Rodríguez, R. , Tinahones, F. J. , Fernández‐Aranda, F. , & Casanueva, F. F. (2014). Association of irisin with fat mass, resting energy expenditure, and daily activity in conditions of extreme body mass Index. Int J Endocrinol, 2014, 857270. 10.1155/2014/857270 24864142 PMC4016898

[phy215198-bib-0026] Qiu, S. , Cai, X. , Sun, Z. , Schumann, U. , Zügel, M. , & Steinacker, J. M. (2015). Chronic exercise training and circulating irisin in adults: A meta‐analysis. Sports Medicine, 45(11), 1577–1588. 10.1007/s40279-014-0293-4 26392122

[phy215198-bib-0027] Rashti, B. A. , Mehrabani, J. , Damirchi, A. , & Babaei, P. (2019). The influence of concurrent training intensity on serum irisin and abdominal fat in postmenopausal women. Menopausal Review, 18(3), 166–173. 10.5114/pm.2019.90810 PMC6970417 31975984

[phy215198-bib-0028] Rezaeimanesh, D. (2020). Effects of interval training on irisin and insulin resistance in overweight men. Archives of Pharmacy Practice, 11, 6.

[phy215198-bib-0029] Rioux, B. V. , Kuwornu, P. , Sharma, A. , Tremblay, M. S. , McGavock, J. M. , & Sénéchal, M. (2017). Association between handgrip muscle strength and cardiometabolic z‐score in children 6 to 19 years of age: Results from the canadian health measures survey. Metabolic Syndrome and Related Disorders, 15(7), 379–384. 10.1089/met.2016.0147 28759349

[phy215198-bib-0030] Sénéchal, M. , Rempel, M. , Duhamel, T. A. , MacIntosh, A. C. , Hay, J. , Wicklow, B. , Wittmeier, K. , Shen, G. X. , & McGavock, J. M. (2015). Fitness is a determinant of the metabolic response to endurance training in adolescents at risk of type 2 diabetes mellitus. Obes Silver Spring Md, 23(4), 823–832. 10.1002/oby.21032 25755198

[phy215198-bib-0031] Sénéchal, M. , Wicklow, B. , Wittmeier, K. , Hay, J. , MacIntosh, A. C. , Eskicioglu, P. , Venugopal, N. , & McGavock, J. M. (2013). Cardiorespiratory fitness and adiposity in metabolically healthy overweight and obese youth. Pediatrics, 132(1), e85–92. 10.1542/peds.2013-0296 23796736

[phy215198-bib-0032] Shim, Y. S. , Kang, M. J. , Yang, S. , & Hwang, I. T. (2018). Irisin is a biomarker for metabolic syndrome in prepubertal children. Endocrine Journal, 65(1), 23–31. 10.1507/endocrj.EJ17-0260 28904307

[phy215198-bib-0033] Siri, W. E. (1993). Body composition from fluid spaces and density: analysis of methods. 1961. Nutrition, 9(5), 480–491. 8286893

[phy215198-bib-0034] Tsuchiya, Y. , Ando, D. , Goto, K. , Kiuchi, M. , Yamakita, M. , & Koyama, K. (2014). High‐intensity exercise causes greater irisin response compared with low‐intensity exercise under similar energy consumption. Tohoku Journal of Experimental Medicine, 233(2), 135–140. 10.1620/tjem.233.135 24910199

[phy215198-bib-0035] Winn, N. C. , Grunewald, Z. I. , Liu, Y. , Heden, T. D. , Nyhoff, L. M. , & Kanaley, J. A. (2017). Plasma irisin modestly increases during moderate and high‐intensity afternoon exercise in obese females. PLoS One, 12(1), e0170690. 10.1371/journal.pone.0170690 28125733 PMC5268488

[phy215198-bib-0036] World Health Organization . (2014). WHO GROWTH CHARTS FOR Canada ‐ 2 TO 19 YEARS: BOYS ‐ Body mass index‐for‐age percentiles [Internet]. Available from: https://www.dietitians.ca/DietitiansOfCanada/media/Documents/WHO%20Growth%20Charts/Set‐1‐BMI_2‐19_BOYS_EN.pdf

[phy215198-bib-0037] World Health Organization . (2014) WHO GROWTH CHARTS FOR Canada ‐ 2 TO 19 YEARS: GIRLS ‐ Body mass index‐for‐age percentiles [Internet]. Available from: https://www.dietitians.ca/DietitiansOfCanada/media/Documents/WHO%20Growth%20Charts/Set‐1‐BMI_2‐19_GIRLS_EN.pdf

[phy215198-bib-0038] Zhang, R. , Fu, T. , Zhao, X. , Qiu, Y. , Hu, X. , Shi, H. , & Yin, X. (2020). Association of circulating irisin levels with adiposity and glucose metabolic profiles in a middle‐aged chinese population: a cross‐sectional study. Diabetes, Metabolic Syndrome and Obesity: Targets and Therapy, 13, 4105–4112. 10.2147/DMSO.S275878 PMC7642586 33162755

